# Novel Roles for P53 in the Genesis and Targeting of Tetraploid Cancer Cells

**DOI:** 10.1371/journal.pone.0110844

**Published:** 2014-11-07

**Authors:** Batzaya Davaadelger, Hong Shen, Carl G. Maki

**Affiliations:** Department of Anatomy and Cell Biology, Rush University Medical Center, Chicago, Illinois, United States of America; University of Illinois at Chicago, United States of America

## Abstract

Tetraploid (4N) cells are considered important in cancer because they can display increased tumorigenicity, resistance to conventional therapies, and are believed to be precursors to whole chromosome aneuploidy. It is therefore important to determine how tetraploid cancer cells arise, and how to target them. P53 is a tumor suppressor protein and key regulator of tetraploidy. As part of the “tetraploidy checkpoint”, p53 inhibits tetraploid cell proliferation by promoting a G1-arrest in incipient tetraploid cells (referred to as a tetraploid G1 arrest). Nutlin-3a is a preclinical drug that stabilizes p53 by blocking the interaction between p53 and MDM2. In the current study, Nutlin-3a promoted a p53-dependent tetraploid G1 arrest in two diploid clones of the HCT116 colon cancer cell line. Both clones underwent endoreduplication after Nutlin removal, giving rise to stable tetraploid clones that showed increased resistance to ionizing radiation (IR) and cisplatin (CP)-induced apoptosis compared to their diploid precursors. These findings demonstrate that transient p53 activation by Nutlin can promote tetraploid cell formation from diploid precursors, and the resulting tetraploid cells are therapy (IR/CP) resistant. Importantly, the tetraploid clones selected after Nutlin treatment expressed approximately twice as much *P53* and *MDM2* mRNA as diploid precursors, expressed approximately twice as many p53-MDM2 protein complexes (by co-immunoprecipitation), and were more susceptible to p53-dependent apoptosis and growth arrest induced by Nutlin. Based on these findings, we propose that p53 plays novel roles in both the formation and targeting of tetraploid cells. Specifically, we propose that 1) transient p53 activation can promote a tetraploid-G1 arrest and, as a result, may inadvertently promote formation of therapy-resistant tetraploid cells, and 2) therapy-resistant tetraploid cells, by virtue of having higher *P53* gene copy number and expressing twice as many p53-MDM2 complexes, are more sensitive to apoptosis and/or growth arrest by anti-cancer MDM2 antagonists (e.g. Nutlin).

## Introduction

Tetraploid cells contain twice the normal amount of DNA and are rare in most normal tissues. However, tetraploid cells are relatively common in cancer and are thought to contribute to tumor development, aneuploidy, and therapy resistance [1]. Direct evidence for the tumorigenic potential of tetraploid cells was provided by Fujiwara et al. [2] who isolated binucleated, tetraploid mammary epithelial cells from p53-null mice. Remarkably, these cells were more susceptible to carcinogen-induced transformation (soft-agar growth) than diploid counterparts, and the tetraploid cells formed tumors in nude mice while diploid cells did not. Other studies have linked tetraploidy to radiation and chemotherapy resistance. For example, Castedo et al. [3], [4] isolated tetraploid and diploid clones from two human cancer cell lines with wild-type p53. Importantly, tetraploid clones were resistant to radiation and multiple chemotherapy agents compared to diploid counterparts. Finally, there is mounting evidence that aneuploid cancer cells are generated from either asymmetric division or progressive chromosomal loss from tetraploid precursors. Early evidence for this came from studies in premalignant Barrett's esophagus. In these studies, the appearance of tetraploid cells correlated with p53 loss and preceded gross aneuploidy and carcinogenesis [5], [6]. In sum, tetraploid cells can have higher tumorigenic potential, be therapy and radiation-resistant, and be precursors to cancer aneuploidy. It is therefore important to identify how tetraploid cells arise and how they can be targeted for cancer treatment.

P53 is a tumor suppressor and important regulator of tetraploidy [7]. p53 is kept at low levels by MDM2, an E3-ligase that binds p53 and promotes its degradation [8], [9]. DNA damage and other stresses disrupt p53-MDM2 binding, causing p53 levels to increase. Increased p53 stops proliferation by inducing expression of genes that promote G1-arrest (*P21*) or apoptosis (*PUMA, NOXA, BAX*) [10]. Evidence that p53 functions in a “tetraploidy checkpoint” comes largely from studies using microtubule inhibitors (MTIs) that block cells in metaphase. Cells arrested in metaphase by prolonged MTI exposure can eventually exit mitosis and enter a pseudo-G1 state, referred to as tetraploid G1 [11], [12]. Endoreduplication refers to the case in which these tetraploid cells enter S-phase and replicate their DNA, giving rise to high ploidy cells with increasing duplications of the genome (8N, 16N, 32N, etc). Cells lacking p53/p21 are more prone to MTI-induced endoreduplication than wild-type cells [11]–[14]. This supports a p53–p21 dependent tetraploidy checkpoint that blocks S-phase entry by 4N cells.

Tetraploid G1-arrest is typically characterized by depletion of G2/M proteins (e.g. Cyclins A/B, CDC2) and increased expression of G1 arrest proteins (e.g. P53, p21) in 4N cells [15]–[19]. DNA damaging agents e.g. ionizing radiation (IR) activate p53 and arrest cells in G1 and G2-phases. Two early reports found IR caused p53 and p21-dependent depletion of CDC2, Cyclin A, and Cyclin B mRNA and protein [20], [21]. In one case, depletion of these G2/M markers coincided with endoreduplication 1–6 days after treatment [21]. These findings suggested that p53–p21 activation in IR-treated cells could promote a tetraploid G1 arrest followed subsequently by endoreduplication. Nutlin-3a (Nutlin) is a preclinical drug that stabilizes p53 by blocking p53-MDM2 binding. We reported that Nutlin could promote a tetraploid G1 arrest in multiple p53 wild-type cell lines, characterized by depletion of G2/M proteins and increased expression of p53 and p21 in 4N cells [18], [19]. Upon Nutlin removal, p53 and p21 decreased and, in some cases, cells underwent endoreduplication [18]. p53 and p21 were required for both the tetraploid G1 arrest and for endoreduplication after Nutlin removal. Importantly, stable tetraploid clones could be isolated from Nutlin treated cells, and these tetraploid clones were resistant to IR and cisplatin (CP)-induced apoptosis [19]. These studies suggest p53–p21 activated by Nutlin can promote a tetraploid G1-arrest and, when p53 levels decrease (Nutlin removal) the cells may replicate their DNA (endoreduplication) and give rise to tetraploid cells that are IR and CP-resistant. However, a caveat of these previous studies is that they were done in cancer cell lines, which may include a mixture of diploid and tetraploid cells, some of which may be inherently IR/CP-resistant. It is possible that we had isolated IR/CP-resistant tetraploid clones that were already present in the population, or that the tetraploid clones we isolated were derived from diploid cells that were already IR/CP-resistant. Thus, it remained unclear whether transient p53 activation by Nutlin could convert diploid cells into tetraploid cells and, if yes, whether the resulting tetraploid clones would display increased resistance to therapy-induced apoptosis. The current study was initiated to address these questions, and to test the possibility that tetraploid cancer cells may be especially sensitive to MDM2 antagonists.

## Materials and Methods

### Cell Lines and Culture Conditions

p53 wild-type and p53-null HCT116 cells (described in [11] were obtained from Dr. Bert Vogelstein (John Hopkins University). D3 and D8 have been described [22] and are diploid clones that were isolated from p53 wild-type HCT116 cells by limiting dilution. The cells were grown in McCoy's 5A medium with 10% fetal bovine serum (FBS), penicillin (100 U/mL) and streptomycin (100 µg/mL). Cells were plated 24 hours before being treated with Nutlin-3 (10 µmol/L; Sigma), irradiation (10 Gy), Cisplatin (Bedford Laboratory), or as indicated.

### Immunoblotting

Whole cell extracts were prepared by resuspending cell pellets in lysis buffer (150 mM NaCl, 5 mM EDTA, 0.5% Nonidet P-40, 50 mM Tris, pH 7.5), resolved by SDS-PAGE, and transferred to polyvinylidene difluoride membranes (NEN Life Science Products). Antibodies to p21 (H-164), Cyclin A (H-432), Geminin (FL-309), Cdk2 (M2) and p53 (AB-6) were from Santa Cruz Biotechnology; antibodies to Cyclin B1 (V152), CDC2 (POH1), and Tyr-15 Phospho-CDC2 were from Cell Signaling; antibodies to MDM2 (2A10) were from Calbiochem. Antibody to Cyclin E (HE-2) was from BD Pharmingen. Primary antibodies were detected with goat anti-mouse (Pierce) or goat anti-rabbit (Life Technologies) secondary antibodies conjugated to horseradish peroxidase, using Clarity chemiluminescence (Bio-Rad).

### Flow Cytometry Analysis and Live Cell Sorting

For cell cycle analysis, cells were harvested and fixed in 25% ethanol overnight. The cells were then stained with propidium iodide (25 µg/ml; Calbiochem). Flow cytometry analysis was performed on Gallios flow cytometer (Beckman Coulter) and analyzed with CellQuest (Becton Dickinson) and FlowJo 8.7 (Treestar, Inc). For each sample, 10,000 events were collected. For live cell sorting, cells were incubated with Hoechst 33342 (2 µmol/liter; Invitrogen) for 90 min at 37°C and then harvested. Cell sorting was performed based on DNA content using a MoFlo cytometer equipped with a UV excitation laser. The sorted cells were plated at low density.

### MRNA analysis

Quantitative real-time PCR (qRT-PCR) was performed to measure mRNA levels for Cyclin A2, Cyclin B1, CDC2, P21, Bax, Noxa, PUMA, P53R2, Actin in cells that were untreated or treated with Nutlin, Cisplatin, or IR as indicated. The quantitative real-time PCR reaction was run in a 7300 Real Time PCR System (Applied Biosystems, Foster, CA) using SybrGreen PCR master mix, (Applied Biosystems, Foster City, CA) following manufacturer's instructions. Thermocycling was done in a final volume of 20 µL containing 2 µL of cDNA and 400 nmol/L of primers (Primers are listed in Table S1). All samples were amplified in triplicate using the following cycle scheme: 95°C for 2 minutes, 40 cycles of 95°C for 15 seconds and 55°C for 60 seconds. Fluorescence was measured in every cycle and mRNA levels were normalized using the Actin values in all samples. A single peak was obtained for targets, supporting the specificity of the reaction.

### Metaphase Spreads

Cell were incubated with Colcemid (50 ng/µL) (Sigma-Aldrich) for an hour at 37°C, harvested and washed with PBS and hypotonic solution (2 parts of 0.075 M KCl and 1 part of 0.7% NaCitrate). The cells were then fixed in fixative (3 part methanol and 1 part acetic acid) and spread onto a slide. The spreads were examined under a phase contrast microscope.

### FISH Analysis

Cells were harvested and washed with PBS. Then incubated with hypotonic solution (2 parts of 0.075 M KCl and 1 part of 0.7% NaCitrate) for 20mins at 37°C. The cells were then fixed in fixative (3 part methanol and 1 part acetic acid). The slides were prepared and aged at 55°C for 3 mins on a thermobrite. The slides were incubated with 0.05% Pepsin (Fisher #P53-100) for 16 mins at 37°C and then washed with PBS and dehydrated in 70%, 85% and 100% ethanol for 1 minute each at room temperature. Then the probe Vysis LSI TP53 SpectrumOrange/CEP 17 SpectrumGreen (Abbott Molecular Inc # 01N17-020) was added hybridize at 73°C for 5 min and 37 degree for 18 hours. Then the slides were washed with SSC buffer (Gibco #15557-044) with 0.5% NP-40 (Fisher #P53-100) for 2 mins at 73°C and counterstained with DAPI II (Abbott Molecular Inc # 30-804818). Slides were examined under fluorescent microscope.

#### Clonogenic Assay

Cells were plated 24 hours before being untreated or treated with CP, IR, or Nutlin in appropriate dilutions to form 50–100 colonies. After 2–3 weeks colonies were fixed with formaldehyde and stained with 0.05% crystal violet. The colonies were counted and normalized with the plating efficiency of untreated cells.

## Results

### Nutlin promotes a tetraploid G1-arrest in diploid cells

**Figure 1:**
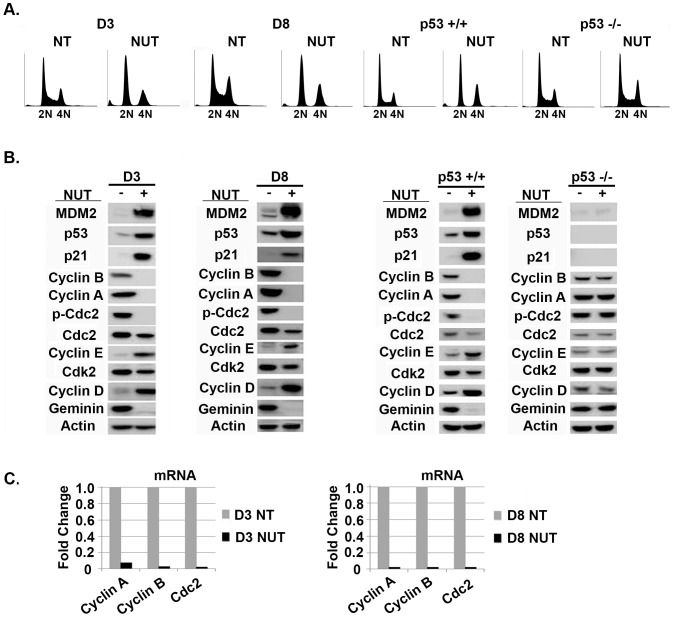
Nutlin-3 causes a p53-dependent tetraploid G1-arrest in diploid HCT116 clones. **A**) HCT116 diploid clones D3 and D8, and HCT116 that express wild-type p53 (p53+/+) or are p53-null (p53-/-) were untreated or treated with Nutlin (NUT 10 µM) for 24 hrs, and cell cycle profile determined by flow cytometry. **B**) Expression of the indicated proteins was monitored by immunoblot in cells untreated or treated with Nutlin (NUT 10 µM) for 24 hrs. **C**) mRNA levels for the indicated factors in untreated cells or cells treated with Nutlin (NUT 10 µM) for 24 hrs was determined by qRT-PCR.

We wished to ask if transient p53 activation by Nutlin could promote tetraploid cell formation from diploid precursors and, if yes, whether the resulting tetraploid cells would have increased resistance to therapy-induced apoptosis. HCT116 is a human colon cancer cell line that expresses wild-type p53. In our previous study, HCT116 were plated at single cell density and ten individual diploid clones were isolated (clones designated D1–D10) that varied in their relative sensitivity to cisplatin (CP)-induced apoptosis [22]. We chose diploid clones D3 and D8 for the current study because these clones showed a relatively high sensitivity to CP in our previous study. D3 and D8 treated with Nutlin for 24 hrs accumulated with 2N and 4N DNA content (Fig 1A). Immunoblots showed that Nutlin treatment in both clones caused complete or near complete depletion of Cyclin A, Cyclin B1, and Tyr-15 phosphorylated CDC2 (pCDC2), and partial depletion of total CDC2 protein (Fig 1B). Cyclin A, Cyclin B1, and CDC2 mRNA were also depleted in Nutlin treated cells, suggesting the decreased level of these G2/M proteins may result from decreased transcription (Fig 1C). In contrast, p53 and p21 protein levels were markedly increased by Nutlin treatment (Fig 1B). Our previous studies showed p53 and p21 were increased in both the 2N and 4N Nutlin-arrested cells [18]. Thus, Nutlin causes depletion of G2/M proteins and increased expression of G1-arrest proteins (p53, p21) in the 4N arrested cells, indicative of a tetraploid G1 arrest. Importantly, Nutlin had no effect on Cyclin A, Cyclin B1, pCDC2, and total CDC2 levels in p53-null HCT116 cells (Fig 1B), and did not cause a 2N and 4N arrest in these cells (Fig 1A), demonstrating the tetraploid G1 arrest induced by Nutlin is p53-dependent. We also monitored levels of geminin, a DNA replication inhibitor that is absent in G1 and accumulates in S, G2, and M-phase [23]. Geminin was depleted by Nutlin in D3 and D8 cells, consistent with the cells being G1-arrested (Fig 1B). Cdk2 levels were unchanged by Nutlin, while levels of G1-phase cyclin proteins Cyclin D1 and Cyclin E were increased, also consistent with cells being arrested in G1-phase. In sum, the results demonstrate that Nutlin causes a p53-dependent tetraploid G1-arrest in diploid HCT116 clones D3 and D8.

### Transient Nutlin treatment promotes formation of therapy resistant tetrapolid clones from diploid precursor cells

**Figure 2:**
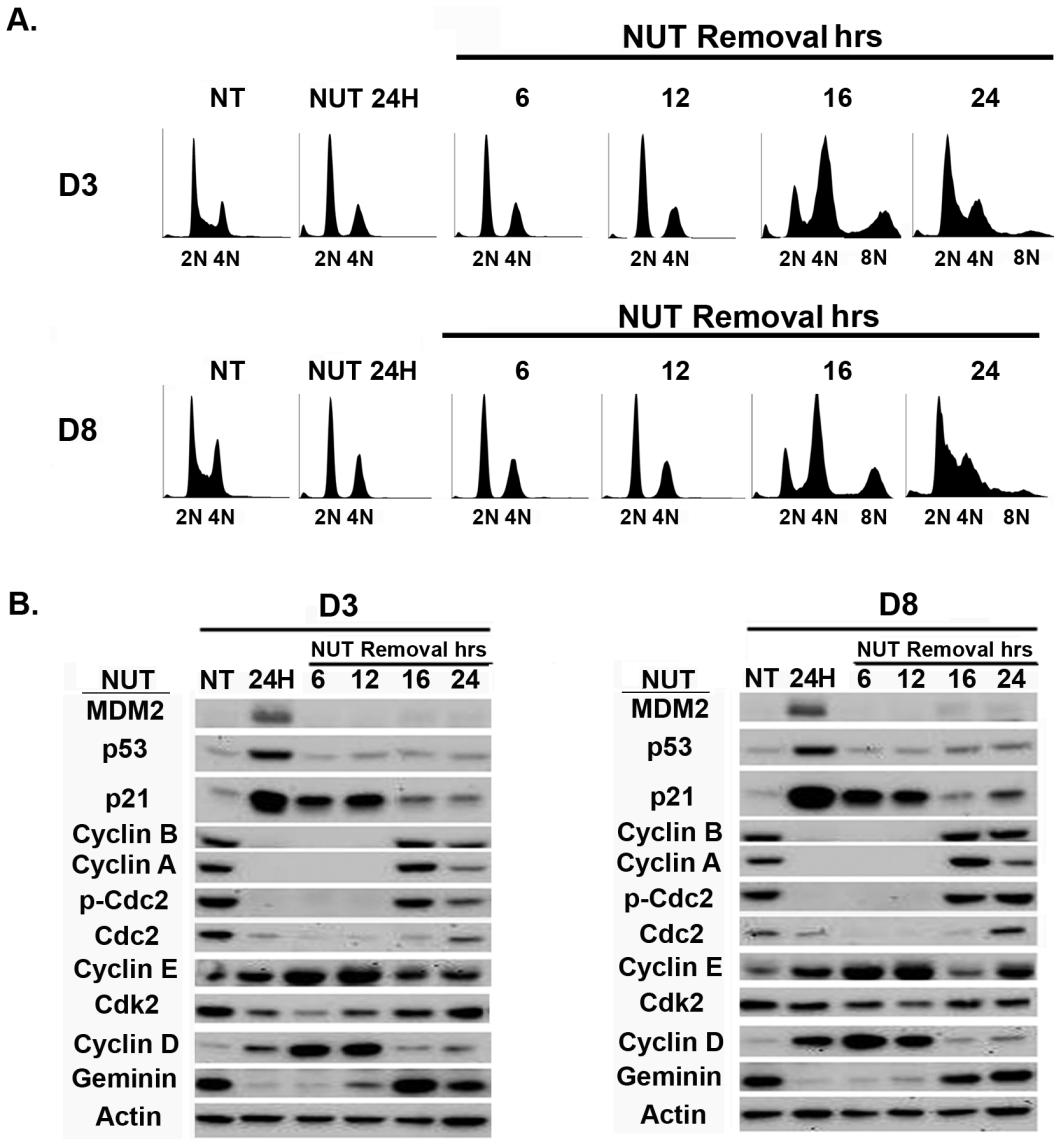
Transient Nutlin-3 treatment of diploid HCT116 clones induces the appearance of cells with>4N DNA content. **A**) Diploid HCT116 clones D3 and D8 were untreated (NT) or exposed to Nutlin (NUT 10 µM) for 24 hrs, followed by Nutlin removal. The cells were harvested at the indicated times after Nutlin removal. Fixed cells were stained with propidium iodide (25 µg/ml) and subjected to flow cytometry analysis. **B**) Cells were untreated (NT) or exposed to Nutlin (NUT 10 µM) for 24 hrs, followed by Nutlin removal. Cell lysates were collected at the indicated time points and analyzed by immunoblotting with the indicated antibodies. Actin was as a loading control. *P-Cdc2*, phosphor-Cdc2 (Tyr-15).

We previously showed 4N arrested cells can reinitiate DNA synthesis after Nutlin removal and replicate their DNA without an intervening mitotic division, a process known as endoreduplication [18], [19]. To ask if D3 and D8 undergo endoreduplication after Nutlin removal, the cells were Nutlin treated for 24 hrs, followed by Nutlin removal for various time points. Flow cytometry was used to monitor cell cycle profiles and immunoblots used to monitor protein levels after Nutlin removal (Fig 2). D3 and D8 accumulated with 2N and 4N DNA content when Nutlin treated for 24 hrs, as in Fig 1. Both clones underwent endoreduplication after Nutlin removal, evidenced by the pronounced emergence of >4N cells 16 hrs after Nutlin removal and their transient accumulation in an 8N peak. The emergence of >4N cells and their accumulation in an 8N peak is indicative of 4N cells initiating DNA synthesis, replicating their DNA, and entering mitosis. Cyclin A2, Cyclin B1, and CDC2 levels returned 16 hrs after Nutlin removal, consistent with the cells being mostly in G2/M at this time point. Cyclin E-CDK2 activity is believed to promote or be required for DNA synthesis initiation [24], [25], and p21 binds to and inhibits Cyclin E-CDK2 activity [26]. Notably, the emergence of >4N, DNA replicating cells 12–16 hrs after Nutlin removal coincided with a sharp decrease in p21 protein levels. We speculate the decreased levels of p21 after Nutlin removal allowed activation of Cyclin E-CDK2 complexes that could then drive DNA synthesis.

**Figure 3:**
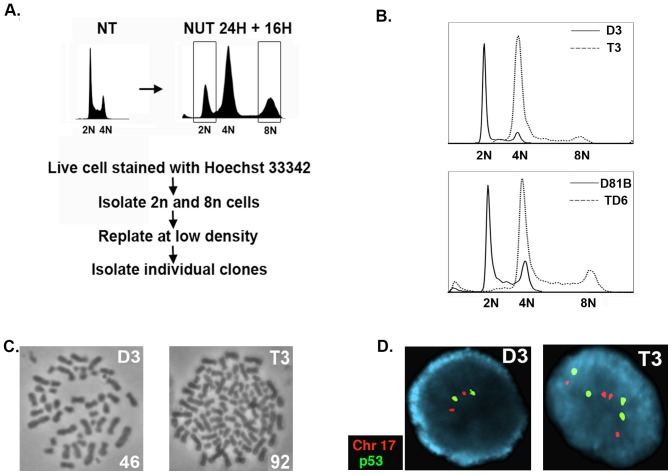
Stable tetraploid clones were isolated from diploid D3 and D8 clones after endoreduplication. **A**) Shown is the procedure to isolate diploid and tetraploid clones from cells transiently exposed to Nutlin. D3 and D8 were untreated (NT) or treated with 10 µM Nutlin (NUT) for 24 hrs, followed by Nutlin removal for an additional 16 hrs. The cells were then live-stained with Hoechst 33342 (5 µg/ml). Cell sorting was performed on a MoFlo cytometer equipped with a UV excitation wavelength laser. Sorted 2N and 8N cells were plated at low density in normal medium (minus Nutlin) and individual clones isolated. **B**) *Top*, the comparison of the DNA profiles between D3 and a representative tetraploid clone isolated from D3. *Bottom*, comparison of the DNA profiles between a representative diploid clone (D81B) isolated from D8 cells, and a representative tetraploid clone (TD6) isolated from D8 cells. **C**) Representative metaphase spread from diploid D3 cells and tetraploid T3 cells. The number in the bottom right indicates the number of chromosomes counted. **D**) FISH analysis with chromosome 17 (Chr 17) and p53-specific probes shows tetraploid (T3) cells contain 4 copies of p53 and Chr 17, while diploid (D3) cells contain 2 copies of p53 and Chr 17.

**Figure 4:**
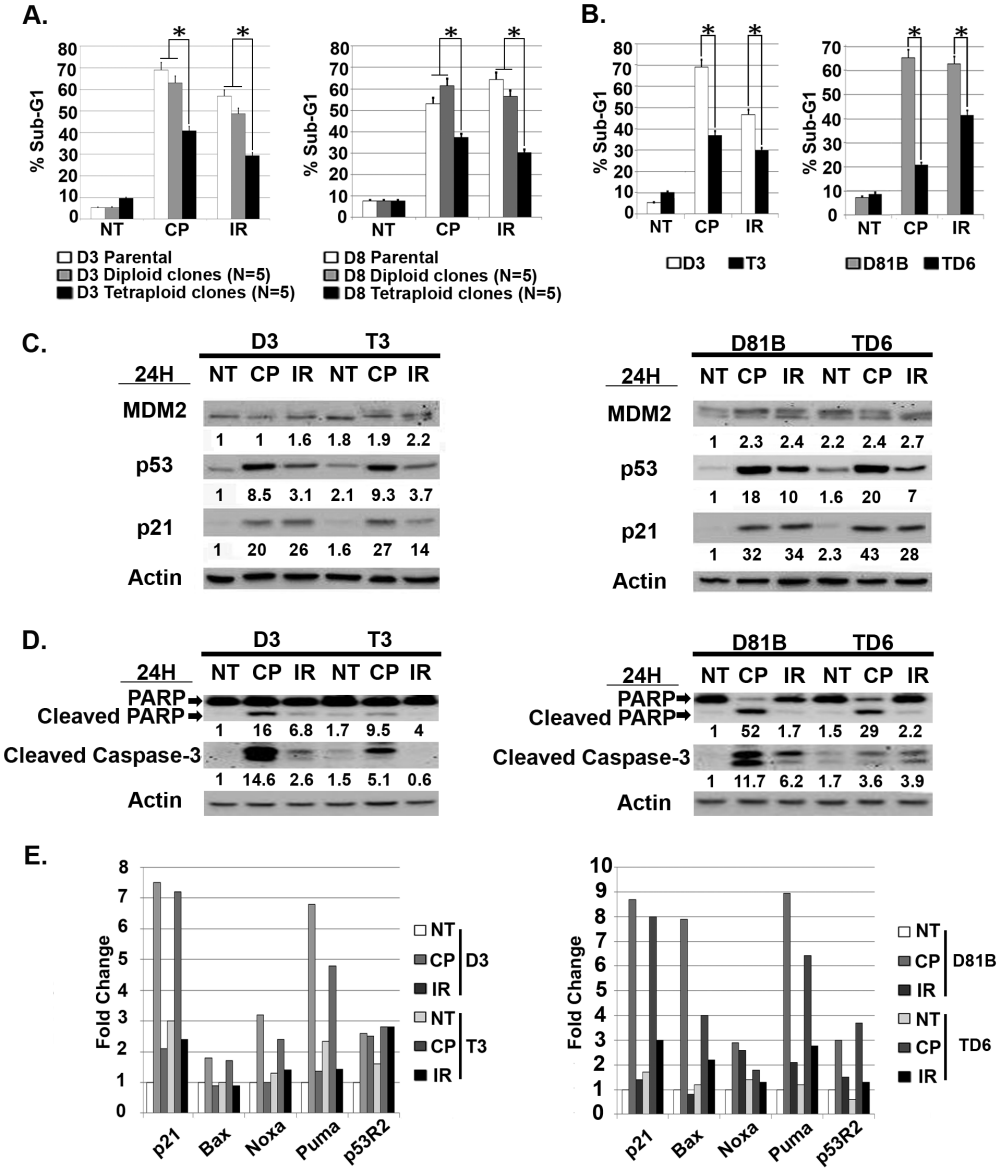
Tetraploid clones show resistance to cisplatin (CP) and ionizing radiation (IR)-induced apoptosis. **A**) Five diploid clones isolated from Nutlin treated D3 cells (D3 Diploid) and D8 cells (D8 Diploid), and five tetraploid clones isolated from Nutlin treated D3 cells (D3 Tetraploid) and D8 cells (D8 Tetraploid) were untreated (NT) or exposed to CP (20 µM) or IR (10 Gy) for 48 hrs. The percentage of cells with sub-G1 DNA was determined. Shown are the mean results from three separate experiments, *bars*, Standard error (SE). * significance value (P<0.05). **B**) Tetraploid clones (T3, TD6) and their diploid counterparts (D3, D81B) were untreated (NT) or exposed to CP (20 µM) or IR (10 Gy) for 72 hrs. The percentage of cells with sub-G1 DNA (propidium iodide staining) was determined. Shown are the mean results from three separate experiments, *bars*, Standard error (SE). * significance value (P<0.05). **C**) The indicated diploid and tetraploid clones were untreated (NT) or exposed to CP (20 µM) or IR (10 Gy) for 24 hrs. p53, p21, and MDM2 protein levels were determined by immunoblotting and quantified. Numbers indicate the relative level of each protein. Actin was used as a loading control. **D**) The indicated diploid and tetraploid clones untreated (NT) or exposed to CP (20 µM) or IR (10 Gy) for 48 hrs. Cleaved PARP and Caspase-3 protein levels were determined by immunoblotting and quantified relative to the untreated. **E**) qRT-PCR was used to determine mRNA levels for the indicated genes in diploid (D3, D81B) and tetraploid (T3, TD6) clones that were either untreated (NT) or exposed to CP (20 µM) or IR (10 Gy) for 24 hrs. The level of each mRNA transcript in untreated diploid clones (D3 NT, D81B NT) was considered “1.0”, and all other values are plotted relative to it.

Next, we wished to ask if endoreduplication after Nutlin removal could give rise to stable tetraploid clones and, if yes, whether the resulting tetraploid clones would be resistant to CP or ionizing radiation (IR)-induced apoptosis. To this end, D3 and D8 were Nutlin treated for 24 hrs followed by Nutlin removal for an additional 16 hrs. The cells were then labeled with the live-cell DNA stain Hoechst 33342. 2N and 8N cells were isolated by flow cytometry and replated at low density for isolation of individual clones (Fig 3A). A total of 11 D3 clones and 12 D8 clones were obtained from isolated 8N populations that arose after Nutlin removal. 7 of the 11 D3 clones (63%) and 8 of the 12 D8 clones (66%) grew as tetraploid 4N cells. This was evidenced by: 1) the G1 peak of these clones overlapping the G2/M peak of the D3 and D8 diploid cells (Fig 3B), 2) chromosome counting of metaphase spreads which supported the tetraploid clones having twice the DNA content of the diploid D3 and D8 precursors (Fig 3C), and 3) FISH analysis using *P53* and chromosome 17-specific probes. This FISH analysis showed tetraploid clones have 4 copies of chromosome 17 and *P53*, while diploid D3 and D8 cells have 2 copies of chromosome 17 and *P53* (Fig 3D). Finally, we tested whether tetraploid clones that arose after Nutlin treatment were more resistant to CP and IR-induced apoptosis than diploid counterparts. First, 5 tetraploid clones and 5 diploid clones isolated from Nutlin treated D3 or D8 cells were exposed to CP (20 µM) or IR (10 Gy), and apoptosis monitored 48 hrs later by sub-G1 DNA content. As shown in Fig 4A, the tetraploid clones as a group were significantly more resistant to CP and IR-induced apoptosis than parental cells and diploid clones isolated after Nutlin treatment. Individual tetraploid clones (T3 and TD6) were also more resistant to CP and IR-induced apoptosis compared to diploid counterparts (D3 and D81B), evidenced by a lower percent sub-G1 cells after CP and IR treatment (Fig 4B) and lower expression of cleaved PARP and cleaved caspase-3 (Fig 4D). These results are consistent with reports by us and others that showed tetraploid cells may be therapy resistant [3], [19]. Previous studies have reported that p53 and p21 can contribute to CP and IR-resistance in HCT116 and other cells, most likely by inducing or enforcing a cell cycle arrest that blocks CP or IR-treated cells from proliferating and attempting to divide [27]–[31]. Notably, we found p53, MDM2, and p21 proteins were induced to comparable levels in CP and IR-treated diploid and tetraploid clones (Fig 4C), and that p53-responsive cell cycle arrest genes (*P21*), DNA repair genes (*P53R2*), and apoptotic genes (*PUMA, Noxa, Bax*), were also induced comparably in the CP and IR-treated diploid and tetraploid clones (Fig 4E). These results suggest CP and IR-resistance in the tetraploid clones is not associated with increased p53 levels or activity. In total, the results indicate transient p53 activation by Nutlin can promote a tetraploid G1 arrest and endoreduplication after Nutlin removal in diploid precursor cells, leading to the generation of therapy (CP/IR)-resistant tetraploid clones.

### Tetraploid clones are more susceptible to p53-dependent apoptosis and growth arrest induced by Nutlin

**Figure 5:**
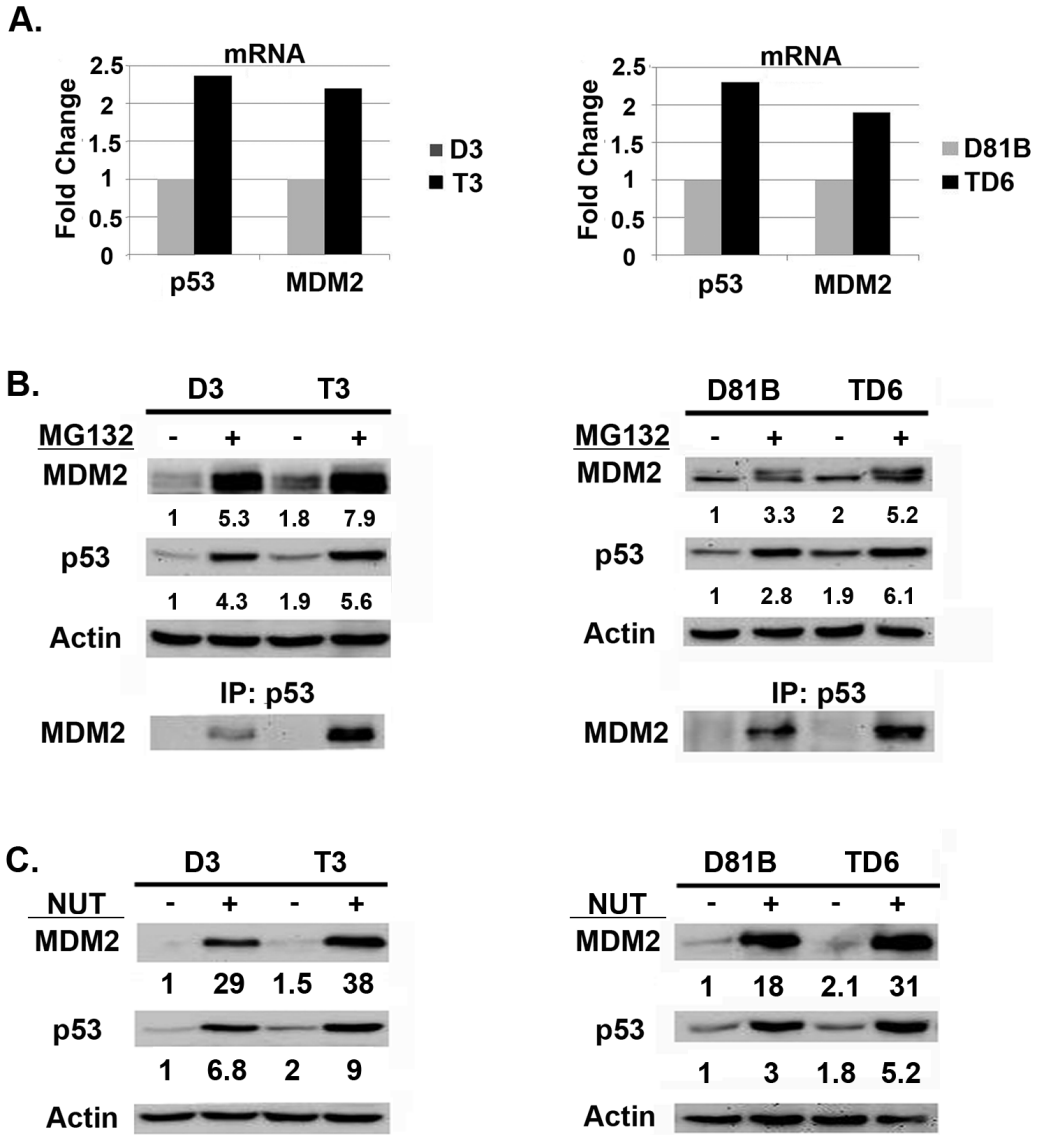
Tetraploid clones express more p53-MDM2 complexes and more p53 after Nutlin treatment than diploid counterparts. **A**) P53 and MDM2 mRNA levels were compared in untreated tetraploid clones (T3, TD6) and their diploid counterparts (D3, D81B). **B**) Diploid (D3, D81B) and tetraploid (T3, TD6) clones were untreated or treated with proteasome inhibitor MG132 (10 µM) for 8 hrs. *Upper* Levels of MDM2, p53, and actin (loading control) in untreated and MG132 treated cells are shown. *Lower* To determine levels of p53-MDM2 complexes in diploid and tetraploid cells, protein lysates were immunoprecipitated with anti-p53 antibody, followed by immunoblotting for MDM2. **C**) Diploid (D3, D81B) and tetraploid (T3, TD6) clones were untreated or treated with Nutlin (10 µM) for 24 hrs. p53 and MDM2 protein levels were detected by immunoblotting and quantified using Image-J software. The relative amount of p53 and MDM2 protein in the untreated diploid clones was given a value of “1.0”. Numbers indicate the relative level of each protein. Actin was used as a loading control.

**Figure 6:**
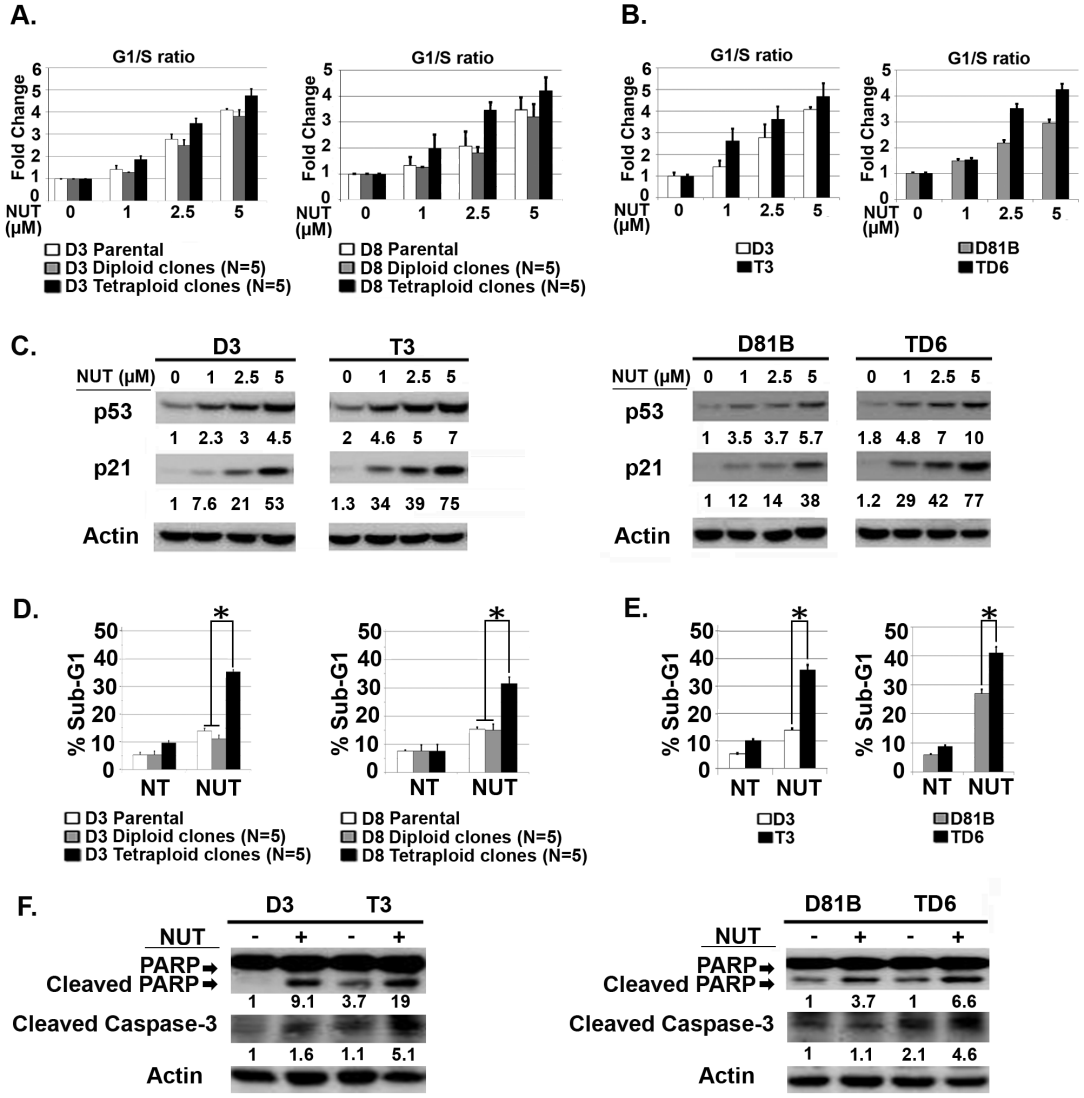
Tetraploid clones are more susceptible to Nutlin-induced cell cycle arrest and apoptosis. **A**) Five diploid clones isolated from Nutlin treated D3 cells (D3 Diploid) and D8 cells (D8 Diploid), and five tetraploid clones isolated from Nutlin treated D3 cells (D3 Tetraploid) and D8 cells (D8 Tetraploid) were untreated or treated with increasing Nutlin (1.0–5.0 µM) for 24 hrs. The percent G1 and S-phase cells was determined by flow cytometry, and the fold change of G1/S ratio is plotted. Untreated diploid clones G1/S ratio was given a value of 1.0. Shown is the average of three separate experiments, +/- SE. **B**) Tetraploid clones (T3, TD6) and diploid counterparts (D3, D81B) were untreated or treated with Nutlin (1.0–5.0 µM) for 24 hrs. The fold change of G1/S ratio is plotted. Shown is the average of three separate experiments, +/- SE. **C**) Diploid (D3, D81B) and tetraploid (T3, TD6) clones were untreated or treated with increasing Nutlin (1.0–5.0 µM) 24 hrs. P53 and p21 protein levels were quantified using Image-J software. Numbers indicate the relative levels of each protein. P53 and p21 levels in untreated diploid clones was given a value of “1.0”. Actin was used as loading control. **D**) Five diploid clones isolated from Nutlin treated D3 cells (D3 Diploid) and D8 cells (D8 Diploid), and five tetraploid clones isolated from Nutlin treated D3 cells (D3 Tetraploid) and D8 cells (D8 Tetraploid) were untreated (NT) or treated with Nutlin (20 µM) 72 hrs and apoptosis determined. Shown are the mean results from three separate experiments, *bars*, Standard error (SE). *(P<0.05). **E**) Representative diploid (D3, D81B) and tetraploid (T3, TD6) clones were untreated (NT) or treated with Nutlin (20 µM) for 72 hrs. The percentage of cells with sub-G1 DNA content (propidium iodide staining) was determined. Shown are the mean results from three separate experiments, *bars*, Standard error (SE). * significance value (P<0.05). **F**) The indicated diploid and tetraploid clones were untreated (NT) or exposed to Nutlin (20 µM) for 48 hrs. Cleaved PARP and Caspase-3 protein levels were determined by immunoblotting and quantified relative to the untreated.

It was somewhat surprising that p53 was induced comparably by CP and IR in diploid and tetraploid clones, despite the fact that the tetraploid clones harbor twice as many copies of the *P53* gene (FISH, Fig 3D). This indicates the level to which p53 is induced by CP and IR is not dependent on *P53* copy number, but instead may be limited by other factors, perhaps the amount of damaged DNA or the activation level of stress-induced kinases that stabilize p53. Nevertheless, we speculated the level to which p53 is induced by MDM2 antagonists (e.g. Nutlin) may depend on the *P53* copy number, and that tetraploid cells may therefore express higher p53 levels in response to Nutlin and be hyper-sensitive to Nutlin-mediated growth inhibition. To test this, diploid D3 and D81B and their tetraploid counterparts T3 and TD6 were Nutlin treated for 24 hrs. P53 and MDM2 protein levels were determined by immunoblot and mRNA levels determined by qRT-PCR. The results showed that the tetraploid clones express elevated (∼2X more) p53 and MDM2 mRNA and protein than diploid clones, both basally (Fig 5A) and after 24 hr Nutlin treatment (Figs 5C). To assess p53-MDM2 complex levels, cells were treated with proteasome inhibitor MG132 to block p53 degradation, and p53-MDM2 complexes determined by co-immunoprecipitation. These studies showed tetraploid clones had more p53-MDM2 complexes after MG132 treatment than the diploid clones from which they arose (Fig 5B). Thus, tetraploid clones express higher levels of p53 and MDM2, and contain higher levels of p53-MDM2 complexes. We speculated in tetraploid clones this would translate to higher p53 levels after Nutlin treatment, and a consequent increase in p53-dependent G1 arrest and apoptosis. To test this, we compared the relative sensitivity of diploid and tetraploid clones to Nutlin-induced cell cycle arrest or apoptosis. First, diploid and tetraploid clones were treated with increasing doses of Nutlin (1.0–5.0 µM) for 24 hrs. Increased G1/S ratio, reflecting depletion of S-phase cells and an accumulation of cells in G1-phase, was used as a measure of Nutlin-induced G1 arrest. As shown in Fig 6A, the tetraploid clones as a group were more susceptible to Nutlin-induced G1 arrest than parental cells or diploid clones (G1/S ratio increased more with increasing Nutlin doses in tetraploid clones than parental or diploid clones). Individual tetraploid clones T3 and TD6 were also more susceptible to Nutlin-induced G1 arrest than diploid counterparts D3 and D81B (Fig 6B). As shown in Fig 6C, p53 and p21 protein were induced to higher levels and at lower Nutlin doses in the tetraploid clones compared to the diploid clones. These results indicate the tetraploid clones express higher levels of p53 and p21 after Nutlin treatment and are more susceptible than diploid clones to Nutlin-induced G1 arrest. Next, diploid and tetraploid clones were treated with a higher Nutlin dose (20 µM) for 72 hrs, and apoptosis monitored by percent cells with sub-G1 DNA content and/or expression of cleaved PARP and cleaved caspase-3. As shown in Fig 6D, tetraploid clones as a group were more susceptible to Nutlin-induced apoptosis than diploid clones. Individual tetraploid clones (T3 and TD6) were also more susceptible to Nutlin-induced apoptosis than diploid counterparts (D3 and D81B), evidenced by a higher percent sub-G1 cells after Nutlin treatment and increased expression of cleaved PARP and cleaved caspase-3 (Figs 6E and F).

**Figure 7:**
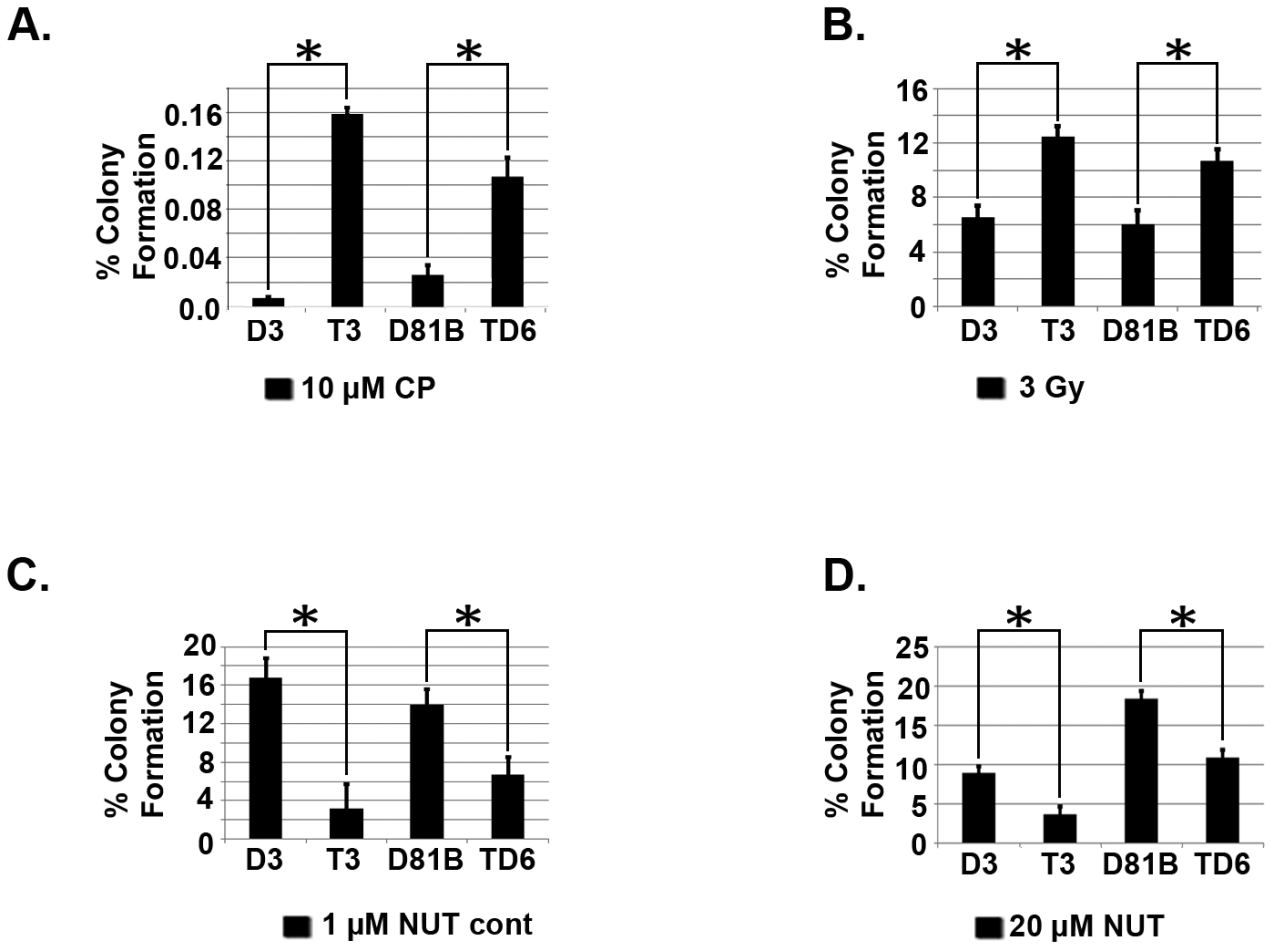
Tetraploid clones are more resistant than diploid clones to Cisplatin (CP) and ionizing radiation (IR) and more sensitive to Nutlin in a long term survival assay. **A**) Diploid (D3, D81B) and tetraploid (T3, TD6) clones were untreated or treated with CP (10 µM) for 24 hrs. Cells were rinsed and re-fed with drug free media and stained after 2–3 weeks. The colonies were counted and normalized with plating efficiency of untreated controls. Shown are the mean results from three separate experiments. **B**) Indicated clones were untreated or exposed to IR (3 Gy). Colonies were counted 2–3 weeks later and normalized with plating efficiency of untreated controls. Shown are the mean results from three separate experiments. **C**) Diploid and tetraploid clones were grown in normal medium (minus Nutlin) or grown in continuous Nutlin (1 µM) and stained after 2–3 weeks. Colony number was normalized with the plating efficiency of untreated controls. **D**) Indicated clones were untreated or treated with Nutlin (20 µM) for 72 hrs after which the cells were rinsed and re-fed with normal medium (minus Nutlin) for 2–3 weeks. The colonies were then counted and normalized with the plating efficiency of untreated controls. Shown are the mean results from three separate experiments. *bars* indicate standard error (SE). * significance value (P<0.05).

Finally, we carried out clonogenic survival assays to complement the apoptosis studies. As shown in Figs 7A and B, tetraploid clones (T3, TD6) showed significantly higher colony forming ability after CP and IR treatment than their diploid counterparts (D3, D81B). In contrast, the tetraploid clones showed lower colony forming ability than diploid counterparts when exposed continuously to 1 µM Nutlin (Fig 7C) or when exposed transiently to 20 µM Nutlin for 3 days (Fig. 7D). The results demonstrate the tetraploid clones are more resistant than diploid counterparts to CP and IR, and more sensitive than diploid counterparts to Nutlin, in both apoptosis and colony formation assays.

## Discussion

Tetraploid (4N) cells contain twice the DNA content of diploid (2N) cells. Tetraploid cells are considered important in cancer because they can display increased tumorigenicity, resistance to conventional therapies, and are believed to be precursors to whole chromosome aneuploidy [1]–[6]. It is therefore important to determine how tetraploid cancer cells arise, and how to target them. Nutlin-3a (Nutlin) is a small molecule MDM2 antagonist and activator of p53. In our previous studies, transient (24 hr) Nutlin treatment promoted a tetraploid G1 arrest in multiple cancer cell lines that express wild-type p53 [18], [19]. This tetraploid G1 arrest was characterized by depletion of G2/M proteins (e.g. Cyclins A/B, CDC2) and increased expression of G1-arrest proteins (e.g. p53, p21) in 4N cells. Upon Nutlin removal, 4N cells reinitiated DNA synthesis and replicated their DNA without an intervening mitosis, a process known as endoreduplication [18], [19]. Both the tetraploid G1 arrest and endoreduplication after Nutlin removal were dependent on p53 and p21. Finally, stable tetraploid clones could be isolated from Nutlin treated cells, and these cells were resistant to ionizing radiation (IR) and cisplatin (CP)-induced apoptosis [19]. These findings suggested transient p53 activation by Nutlin can promote endoreduplication and the generation of therapy resistant tetraploid cells. However, a caveat is that these previous studies were done in cancer cell lines, which could include a mixture of diploid and tetraploid cells, some of which may be inherently IR/CP-resistant. It is possible that we had isolated IR/CP-resistant tetraploid clones that were already present in the population, or that the tetraploid clones we isolated were derived from individual diploid cells that were already IR/CP-resistant. Therefore, in the current report we asked if Nutlin could promote tetraploid cell formation from diploid precursors and, if yes, whether the resulting tetraploid cells would show increased resistance to IR and/or CP. Individual diploid clones (D3, D8) isolated from the HCT116 colon cancer cell line by limiting dilution were treated with Nutlin for 24 hrs, followed by Nutlin removal. Nutlin promoted a tetraploid G1 arrest in the diploid clones that was p53-dependent. The clones underwent endoreduplication after Nutlin removal, giving rise to stable tetraploid clones that showed increased resistance to IR/CP-induced apoptosis compared their diploid counterparts. These findings demonstrate that transient p53 activation by Nutlin can promote tetraploid cell formation from diploid precursors, and the resulting tetraploid cells are therapy (IR/CP) resistant. Importantly, tetraploid clones selected after Nutlin treatment expressed twice as much *P53* and *MDM2* mRNA as diploid cells, expressed twice as many p53-MDM2 protein complexes (by co-immunoprecipitation), and were more susceptible to p53-dependent apoptosis and growth arrest induced by Nutlin. Based on these findings, we propose that p53 plays a role in both the formation and targeting of tetraploid cells. Specifically, we propose that 1) transient p53 activation can promote a tetraploid-G1 arrest and, as a result, may inadvertently promote formation of therapy-resistant tetraploid cells, and 2) therapy-resistant tetraploid cells, by virtue of having higher *P53* gene copy number and expressing twice as many p53-MDM2 complexes, are more sensitive to apoptosis and/or growth arrest by anti-cancer MDM2 antagonists (e.g. Nutlin).

In order for endoreduplication to occur, 4N (G_2_ or M phase) cells must first assume a G_1_-like state from which they can enter S phase, referred to as tetraploid G_1_. Typically, tetraploid G_1_ is characterized by depletion/loss of G_2_/M marker proteins (*e.g.* Cyclins A/B, CDC2) and increased expression of G_1_ phase markers in 4N cells [15]–[19]. Diploid HCT116 clones treated with Nutlin for 24 hrs arrested with 2N and 4N DNA content, coincident with increased expression of p53 and p21 protein, and complete or near-complete depletion of Cyclin A, Cyclin B, and CDC2. We previously showed p53 and p21 are expressed in both the 2N and 4N Nutlin-arrested cell pools [18]. Thus, Nutlin causes depletion of G2/M proteins and increased expression of G1-arrest proteins (p53, p21) in 4N cells, indicative of a tetraploid G1 arrest. We previously showed p53 and p21 are required for depletion of Cyclins A/B and CDC2 protein by Nutlin [18], [19], and in the current study we also observed mRNA levels for Cyclin A, Cyclin B, and CDC2 are drastically reduced in Nutlin treated cells. This suggests the decrease in Cyclin A, Cyclin B, and CDC2 protein levels may result from decreased gene transcription. The Cyclin A, Cyclin B, and CDC2 gene promoters harbor E2F binding sites and are E2F-responsive [32]. pRb proteins (pRb, p107, p130) can block expression of E2F responsive genes by binding and sequestering E2F proteins away from the promoter, or by forming transcription repressor complexes with E2F on DNA gene promoters [32]. Active Cyclin-CDK complexes can phosphorylate pRb proteins, blocking their interaction with E2Fs. In contrast, p21 activates pRb proteins by binding and inhibiting the activity of Cyclin-CDKs [26]. Thus, one possibility is that p53–p21 induced by Nutlin activates pRb, p107, and/or p130, which may then bind E2F complexes and inhibit Cyclins A/B and CDC2 mRNA and protein expression, contributing to a tetraploid G1-arrest.

A second requirement for endoreduplication is that DNA replication origins are “licensed” in tetraploid G1 cells. Origin “licensing” involves the sequential binding of the origin recognition complex (ORC), CDC6 and Cdt1, and the replicative helicase MCM2-7 [33]. Geminin binds and inhibits Cdt1, preventing assembly of pre-replication origin licensing complexes [23]. Geminin is normally expressed in S, G2, and M-phases, but not expressed in G1 when origin licensing occurs [23]. We observed a pronounced depletion of geminin in Nutlin-arrested 2N and 4N cells, consistent with the cells being arrested in a G1 state. Notably, the geminin gene promoter is also E2F-responsive [34]. Thus, geminin expression may also be reduced in Nutlin treated cells at the mRNA level, resulting from p21 activation of pRb/p107/p130 and inhibition of E2F, as described above. Given that geminin is depleted in Nutlin treated cells, we speculate that pre-replicative origin licensing complexes are formed in the 2N and 4N Nutlin-arrested cells. Subsequent origin firing/S-phase entry occurs upon recruitment of the DNA replication machinery and activation of Cyclin E-CDK2 [24], [25]. In the current study, endoreduplicating ( >4N) cells emerged 12–16 hrs after Nutlin removal, coincident with a sharp decrease in p21 protein levels. We speculate the most likely scenario is that the reduction in p21 levels allowed activation of Cyclin E-CDK2 complexes, which then triggered origin firing/S-phase entry by 4N cells.

Tetraploid clones that arose after Nutlin treatment were resistant to IR and CP compared to the diploid cells from which they came. Given that the Nutlin effects are p53-dependent, the results demonstrate that transient p53 activation can promote formation of therapy resistant tetraploid cells from diploid precursors. The basis for IR/CP-resistance in the tetraploid cells is unclear. Castedo et al. compared radiation and chemosensitivity of diploid RKO and HCT116 cells with tetraploid clones that arose after prolonged nocodazole treatment [3], [4]. Tetraploid clones were significantly more resistant to IR, CP, and other agents. In their study, tetraploid clones expressed increased basal and therapy-induced levels of P53R2, a p53-responsive ribonucleotide reductase DNA repair enzyme [3], [4]. Knockdown of P53R2 in their study sensitized tetraploid clones to apoptosis by IR and other agents. In the current study, we found P53R2 was expressed comparably at the mRNA level in the diploid and tetraploid clones either basally or after IR or CP treatment, suggesting IR and CP resistance in the tetraploid clones in our study is not associated with increased P53R2 expression. Notably, p53-null cells are sometimes more sensitive than p53 wild-type HCT116 cells to IR and CP-induced killing, probably because p53 can activate/enforce cell cycle arrests that block IR/CP treated cells from continuing to replicate with damaged DNA [27]–[31]. Given they have twice as many *P53* gene copies, we considered tetraploid clones might express more p53 protein than diploid cells after IR or CP treatment and therefore be more resistant. However, we found p53 was induced to a comparable level in IR and CP-treated diploid and tetraploid cells. Thus, the increased IR and CP-resistance in tetraploid cells in our study does not appear to be associated with increased p53 levels or activity.

Targeting tetraploid cancer cells is an important goal given the potential involvement of these cells in tumor progression, therapy resistance, and aneuploidy. The tetraploid clones that arose after Nutlin treatment in the current study contained twice as many *P53* gene copies (by FISH), expressed approximately twice as much *P53* and *MDM2* mRNA as diploid cells, and expressed approximately twice as many p53-MDM2 protein complexes (by p53 and MDM2 co-immunoprecipitation). We speculated that if tetraploid cells contain more p53-MDM2 complexes than diploid cells, they might be hypersensitive to MDM2 antagonists that disrupt these complexes. Indeed, we found p53 (and p21) were induced to a higher level in Nutlin-treated tetraploid cells vs Nutlin-treated diploid cells, and the tetraploid cells were more sensitive than diploid cells to Nutlin in apoptosis, colony formation, and cell cycle arrest assays. In sum, the results suggest therapy-resistant tetraploid cells, by virtue of having higher *P53* gene copy number and expressing twice as many p53-MDM2 complexes, are more sensitive than diploid cells to anti-cancer MDM2 antagonists (e.g. Nutlin).

## Conclusions

Transient p53 activation can promote a tetraploid-G1 arrest and, as a result, may inadvertently promote formation of therapy-resistant tetraploid cells. Therapy-resistant tetraploid cells, by virtue of having higher *P53* gene copy number and expressing twice as many p53-MDM2 complexes, are more sensitive to apoptosis and/or growth arrest by anti-cancer MDM2 antagonists (e.g. Nutlin).

## Supporting Information

Table S1 **Primers used for gene expression analysis.**
(DOC)
