# Supplementary material for: Novel Roles for P53 in the Genesis and Targeting of Tetraploid Cancer Cells
Source: PLoS One. 2014 Nov 7;9(11):e110844. doi: 10.1371/journal.pone.0110844 (PMC4224386; doi:10.1371/journal.pone.0110844)
Supplement: Table S1 — Primers used for gene expression analysis. (DOC) [file pone.0110844.s001.doc]

| **Genes** | **Forward Primer** | **Reverse Primer** |
| --- | --- | --- |
| Cyclin A2 | TGGAAAGCAAACAGTAAACAGCC | GGGCATCTTCACGCTCTATTT |
| Cyclin B1 | AATAAGGCGAAGATCAACATGGC | TTTGTTACCAATGTCCCCAAGAG |
| CDC2 | GGATGTGCTTATGCAGGATTCC | CATGTACTGACCAGGAGGGATAG |
| P21 | ACTCTCAGGGTCAGGGTCGAAAACGG | CCTCGCGCTTCCAGGACTG |
| Bax | GCCCTTTTGCTTCAGGGTTT | TCCAATGTCCAGCCCATG |
| Noxa | TGGAAGTCGAGTGTGCTACTCAACT | AGATTCAGAAGTTTCTGCCGGAA |
| Puma | AGAGGGAGGAGTCTGGGAGTG | GCAGCGCATATACAGTATCTTACAGG |
| P53R2 | AGAGGCTCGCTGTTTCTATGG | GCAAGGCCCAATCTGCTTTTT |
| Actin | TCGTGCGTGACATTAAGGAG | GTCAGGCAGCTCGTAGCTCT |

**Supplemental Table 1. Primers used for gene expression analysis.**
